# Adherence to iron supplements among women receiving antenatal care at Mulago National Referral Hospital, Uganda-cross-sectional study

**DOI:** 10.1186/s13104-017-2834-z

**Published:** 2017-10-25

**Authors:** Tusuubira S. Kiwanuka, Sam Ononge, Paul Kiondo, Fatuma Namusoke

**Affiliations:** Department of Obstetrics and Gynaecology, School of Medicine, College of Health Sciences, Makerere University, P. O. Box 7072, Kampala, Uganda

**Keywords:** Adherence, Pregnancy, Iron supplementation

## Abstract

**Background:**

Antenatal iron supplementation is a cost effective way of reducing iron deficiency anaemia among pregnant women in resource limited countries like Uganda. Poor adherence to iron supplements has limited its effectiveness in reducing maternal anaemia as evidenced by the high burden of iron deficiency anemia in Sub-saharan Africa. The aim of this study was to determine the level of and factors associated with adherence to iron supplementation among women attending antenatal clinic at Mulago National Referral Hospital, Kampala, Uganda.

**Methods:**

Three hundred and seventy pregnant women were recruited in a cross sectional survey in Mulago National Referral Hospital antenatal clinic after informed consent between February and April 2014. Levels of adherence to iron supplements were assessed using visual analogue scale and factors associated collected using an interviewer administered questionnaire.

**Results:**

About 12% (11.6%) of the mothers attending the antenatal clinic adhered to iron supplements over 30 day period. Mothers who had had four or more antenatal visits prior to the survey [odds ratio (OR) = 1.49, 95% confidence interval (CI) 1.12–1.97], had more than 2 week supply of iron supplements in the previous visit (OR 2.81, 95% CI 1.02–1.09), prior health education (OR 1.56, 95% CI 1.07–2.29) were more likely to adhere to iron supplements. Inadequate drug supplies and fear for side effects were the main reasons why participants missed the iron supplements.

**Conclusions:**

There was low adherence to iron supplements among mothers attending antenatal clinic at Mulago National Referral  Hospital. We recommend a national evaluation of adherence to iron supplements and look at ways of increasing adherence.

## Background

Iron deficiency anaemia (IDA) in pregnancy is associated poor maternal and neonatal outcomes like premature labor, post-partum haemorrhage, low birth weight and poor cognitive development [1]. In Uganda, anaemia in pregnancy is estimated to affect about 33% of the pregnancies [2]. To prevent morbidity associated with IDA, the Ministry of Health in Uganda recommends use of daily oral iron and folic acid supplementation during pregnancy and within 6 weeks after delivery [3]. In order to achieve the effectiveness of medication/intervention, it requires up to 80% adherence [4]. Previous studies on iron adherence have had conflicting results, with some reporting adherence of up to 88% [5] but with majority reporting low levels of 25–50% [6–8] which may explain persistence of IDA. There was paucity of data on burden and factors associated with adherence to antenatal iron supplementation in this setting. The purpose of this study was to determine the level of and the factors associated with adherence to iron supplementation among women receiving antenatal care at Mulago National Referral Hospital.

## Methods

### Study setting and participants

This study was a cross sectional study conducted on women in second or third trimesters, receiving antenatal care at Mulago National Referral Hospital from February to April 2014. The hospital serves as a National Referral Hospitals for Uganda and Makerere University College of Health Sciences teaching hospital. Mulago National Referral Hospital is a government institution which gives free health care services. It has two antenatal clinics, one is a high risk clinic run by the obstetricians/gynaecologists, and low risk clinic managed by midwives. The obstetrician led antenatal clinic serves an average of 130 mothers per day and runs 3 days in a week. The midwife led antenatal clinic caters for an average of 60–80 women per day for 5 days. Mulago hospital provides the iron supplements at no cost, however, in cases where the prescribed medicines are not available/inadequate supplies at the unit, the clients are given a prescription to buy from a private pharmacy. During the antenatal visits, mothers receive tetanus immunization, health education talks and are counseled for HIV testing. They are given haematinics and antimalarials for prevention of anaemia and malaria respectively.

The women who had had an antenatal visit 30 days or more prior to recruitment and were eligible for receipt of iron supplements on their previous visit were included. Women were excluded if they were allergic to iron supplements. Sample size for prevalence was calculated using the Kish Leslie formula (1965). Non-compliance of 58% was depicted from a study done in Tanzania to assess adherence for the conventional iron supplements. Adherence of 42% was found for the conventional iron supplements [12]. With power of 80%, and desired level of precision of 5%, the minimum sample size estimated for the study was 374 participants.

### Study procedures

Data on socio demographic characteristics, obstetric history and health system related characteristics was collected using an interviewer administered questionnaire. Information on age of the women, their education level, marital status and occupation was recorded. The occupation of the mother was used as a proxy for the socioeconomic status. Data on the number of antenatal visits the mother had prior to enrollment (ANC visits), total duration of use of iron supplements, number of iron tablets supplied at the hospital pharmacy during the last ANC and getting health talks on usefulness of iron supplements was collected using an interviewer administered questionnaire.

Adherence to antenatal iron supplements in the last 30 days was assessed using the visual analogue scale. Using a scale, patients were asked to self-assess their level of adherence on a scale of 0–100% without being observed by the interviewer. Adherence was assessed as good, partial or poor if patients were taking ≥ 90, 50–90, < 50% of their prescribed pills respectively. For purposes of this analysis, the partially and poorly adherent groups were combined to form the non-adherent group while the group that had good adherence formed the adherent group.

### Statistical methods

The data was entered in Epi-Data software package 3.1 and analysed using STATA version 11. The dependent variable was adherence to iron supplements and independent variables were the patient (age, parity, marital status, total number of antenatal visits, total duration of use of iron supplements during that pregnancy, education level, occupation) and hospital related factors (number of tablets dispensed at the hospital pharmacy during the last ANC visit, health talks on iron supplements). Descriptive statistics was done to describe the participants and then bivariate and multivariate logistic regression using a backward elimination method to determine factors which were independently associated with good adherence. Statistical significance was put at a p value of less than 0.05.

## Results

### Socio-demographic characteristics of the participants

The median age of the study participants was 27 years and the interquartile range was 8 years. Nearly 27% (102/370) of study participants were primigravidae and (244/370) 66% of them had post primary education. The details of the baseline characteristics of the participants are summarized in Table 1.

**Table 1:** Demographic characteristics of study participants

Variable	Number (N = 370)	Percentage (%)
Marital status		
Single	43	11.6
Married	321	86.8
Widowed	4	1.1
Separated/divorced	2	0.5
HIV status		
Negative	332	89.7
Positive	38	10.3
Education status		
Primary and below	126	34.0
O’level and above	244	66.0
Parity		
None	111	30.0
One	75	20.3
2–4	149	40.3
≥ 5	35	9.4
Occupation		
Formal	42	11.4
Informal	144	38.9
None	184	49.7

### Health system related factors

Ninety percent of the study participants, (333/370) reported having received iron supplements on their previous antenatal visit. Over 60% of the participants had attended the antenatal clinic less than four times and 17.6% had been using iron supplements for more than 90 days. Details on the health related factors in the participants are shown in Table 2.

**Table 2:** Health system related factors in relation to adherence

Variable	Adherence (p0)	Non adherence (p1)	Total N (%)	p value (for p0–p1)
Number of ANC visits				
< 4 visits	202 (61.8)	20 (46.6)	222 (60.2)	0.06
≥ 4 visits	124 (38.1)	23 (53.4)	147 (39.8)	0.06
Received iron at previous visit				
Yes	291 (89.0)	42 (97.7)	333 (90.0)	0.07
No	36 (11.0)	1 (2.3)	37 (10.0)	0.07
Counseled on iron before				
Yes	117 (35.8)	25 (58.1)	142 (38.4)	0.00
No	220 (64.2)	18 (41.9)	228 (61.6)	0.00
Duration of use of iron				
≤ 90 days	272 (83.2)	33 (76.7)	305 (82.4)	0.29
> 90 days	55 (16.8)	10 (23.3)	65 (17.6)	0.29
Where do you get drugs from				
Hospital pharmacy	286 (87.5)	38 (88.4)	324 (87.6)	0.87
Private clinic/local store	41 (12.5)	5 (11.6)	46 (13.4)	0.87
Do you always get your drugs				
Yes	247 (75.5)	38 (88.4)	285 (77.0)	0.06
No	80 (24.5)	5 (11.6)	85 (23)	0.06
Iron tablets received on last visit				
≤ 14 tablets	128 (39.2)	8 (18.6)	136 (36.8)	0.01
> 14 tablets	199 (60.8)	35 (81.4)	234 (63.2)	0.01
Are you taking other medication?				
Yes	75 (22.9)	16 (37.2)	91 (24.6)	0.04
No	252 (77.1)	27 (62.8)	279 (75.4)	0.04
Reason for missing				
Side effects	51 (15.6)	0 (0.0)	51 (13.8)	0.00
Too many drugs	5 (1.5)	1 (2.3)	6 (1.6)	0.69
I forgot	90 (27.5)	3 (7.0)	93 (25.1)	0.00
No drugs	129 (39.4)	5 (11.6)	134 (36.2)	0.00
Not important	20 (6.1)	0 (0.0)	20 (5.4)	0.09
Other	32 (9.8)	34 (79.1)	66 (17.8)	0.00

### Very low adherence to iron supplementation in pregnancy

Eighty-two percent (304/370) of the participants reported ever missing to take iron supplements during the current pregnancy and the commonest reason was lack of drugs (134/370) and followed by forgetting to take medication (93/370). Using the VAS, good adherence (taking 90% or more of prescribed iron supplements) over 30 days was 11.6% (43/370), with (203/370) 54.9% having partial adherence and (124/370) 33.5% having poor adherence. The partial and poorly adherent group were combined to form the non-adherent group which constituted (327/370) 88.4% of the study participants. The participants who had had more than four antenatal visits and who received health talks on the usefulness of iron supplements were more likely to adhere. The details on other factors associated with adherence at bivariate analysis are presented in Table 3.

**Table 3:** Factors associated with adherence to iron supplements—bivariate analysis

Variable	Non adherence N (%)	Adherence N (%)	OR	95% CI	p value
Number of ANC visits*					
< 4 ANC visits	202 (61.8)	20 (46.6)	1.0		
≥ 4 ANC visits	125 (38.2)	23 (53.4)	1.87	0.99–3.55	0.05
Received iron at previous visit*					
Yes	291 (89.0)	42 (97.7)	1.0		
No	36 (11.0)	1 (2.3)	0.19	0.02–0.76	0.11
Health talk on supplements*					
Yes	107 (28.9)	25 (58.1)	1.0		
No	220 (64.2)	18 (41.9)	0.40	0.21–0.76	0.01
Duration of use of iron					
≤ 90 days	272 (83.2)	33 (76.7)	1.0		
> 90 days	55 (16.8)	10 (23.3)	1.50	0.69–3.21	0.30
Do you always get your drugs*					
Yes	247 (75.5)	38 (88.4)	1.0		
No	80 (24.5)	5 (11.6)	0.41	0.15–1.07	0.07
Iron tablets received on last visit*					
≤ 14 tablets	128 (39.2)	8 (18.6)	1.0	–	–
> 14 tablets	199 (60.8)	35 (81.4)	2.81	2.26–6.25	0.01
Are you taking other medication?*					
No	75 (22.9)	16 (37.2)	1.0		
Yes	252 (77.1)	27 (62.8)	1.99	1.02–3.89	0.04

*Significant at bivariate analysis

### Individual and institutional factors associated with adherence to iron supplements

Participants who had attended the antenatal clinic four or more times before recruitment (OR 1.49 95% CI 1.12–1.97), receiving a supply of iron tablets of more than 14 days (OR 1.05; 95% CI 1.02–1.09), receiving health education about the supplements (OR 1.56, 95% CI 1.07–2.29). The participants who were in informal employment and unemployed were more likely to adhere to iron supplements (OR 11.80, 95% CI 1.45–96.13). Details on all the factors which remained significant after multivariate analysis are shown in Table 4.

**Table 4:** Factors associated with adherence to iron supplements—multivariate analysis

Variable	Unadjusted OR	Adjusted OR	95% CI	p value
Number of ANC visits				
< 4 visits	1.0	1.0	–	–
≥ 4 visits	1.87	1.49	1.12–1.97	0.01^¶^
Health talk on supplements				
No	1.0	1.0	–	–
Yes	0.40	1.56	1.07–2.29	0.02^¶^
Iron tablets received on last visit				
≤ 14 tablets	1.0	1.0	–	–
> 14 tablets	2.81	1.05	1.02–1.09	< 0.001^¶^
Are you taking other medication?				
Yes	1.0	1.0	–	–
No	1.99	1.37	0.52–3.38	0.48
HIV status				
Negative	1.0	1.0	–	–
Positive	2.72	1.43	0.47–4.38	0.52
Occupation				
Formal employment	1.0	1.0	–	–
Informal employment	7.0	11.80	1.45–96.13	0.02^¶^
Not employed	5.28	10.23	1.25–84.59	0.03^¶^

^¶^Significant at multivariate analysis

## Discussion

Despite the high burden of anaemia in pregnancy in low resource setting and effectiveness of antenatal iron supplementation, we found that only 12% of participants adhered to iron supplements. Similarly very low adherence to iron supplements has been shown in the Scandinavia of 27% [8]. Conversely, higher levels of adherence to iron supplements has been reported in Nigeria and Senegal of 88 and 58% respectively [5, 7]. The adherence to iron supplements in this study was very low compared to that reported in other African countries. It is possible that the adherence levels seen in Senegal were due to the fact that a lower cut off (70%) was used to assess adherence. The differences in levels of adherence can be explained by the different methods used in data collection and definition of adherence in the studies. It has been suggested adherence to medication should be defined as a patients taking 90% or more of prescribed medicine [9]. The general weakness of most studies assessing adherence is the use of self reports in measurement of adherence. The challenge with self reports is that patients tend to overestimate their adherence [9–11]. For example in a study done in Iran, 80% of the pregnant women reported taking their iron supplements but this could only be confirmed in 21% of participants after examining the stool samples [12].

Using the visual analogue scale, about 12% of participants adhered to their iron supplements in this study. VAS is a tool used in data collection of adherence where the participant judges herself/himself on a given scale in absence of the interviewer has been suggested to be reliable and cheap. Previous studies done to assess adherence to ARVs, anti-TB drugs and pain assessment have identified the VAS as a reliable method of assessing adherence in resource limited settings [10, 13]. Other methods like pill count have been used but it is time consuming.

One of the main findings in our study was that mothers were not getting enough iron supplements to last them till the next visit were less likely to adhere to the supplements as prescribed. Galloway et al. [14] in a systemic review identified supplies as the main reason that women don’t comply to their iron supplements. In a study done in Vietnam, supplies were identified as the most important factor affecting adherence [15]. Most women, if provided with adequate iron supplements, whether pills or syrup are likely to take them [14, 16]. The burden of insufficient supplies of iron supplements is still reflected in the USAID cross country report across 33 developing countries with less than 50% of pregnant women having bought or received iron supplements in the preceding pregnancy in the eight African countries studied [4].

Another factor that was independently associated with adherence was providing women with information on importance of iron supplements through health talks. Previous studies have shown that providing mothers with information is one of the ways to improve the effectiveness of iron supplementation programmes [17, 18]. Patients who know why they are taking their medication and how to deal with the possible complications that may arise are likely to adhere to their medications [14, 18, 19]. Health talks during pregnancy are a very good avenue for conveying massages to the pregnant women.

Pregnant women who had attended the antenatal clinic four or more times were more likely to adhere to their iron supplements than their counterparts after controlling all factors. In a cross country survey carried out in 33 developing countries in Africa, Asia and Latin America, there was no statistically significant correlation between the median number of ANC visits that a mother had and the number of days women consumed their iron tablets [16]. Prior studies have shown that the number of antenatal visits had no added advantage to maternal welfare when compared to the four ANC visits recommended by WHO provided there were no maternal complications [20]. The possible explanation is that, women who attended more than four times are likely to have had a complicated pregnancy and therefore adherence issue discussed with physician.

From our study, side effects were one of the main reasons that mothers gave for failing to adhere to their iron supplements. There’s conflicting evidence regarding the impact of side effects on adherence to iron supplements. While some studies show that side effects are actually the main reason why some mothers stop taking their drugs, others argue otherwise. In a study by Nir Melamed to assess the effect of side effects on compliance, it was found that although 45% of the participants reported at least one side effect, only 18.3% attributed discontinuation of therapy to side effects [21]. Different studies that have reviewed iron supplementation programmes show that the proportion of pregnant women who stop taking drugs due to side effects are actually low [14, 16, 18, 21, 22]. In Norway, women receiving a placebo complied as well as those on iron supplements, suggesting that side effects may actually not be important [14]. In Tanzania, women who were taking the conventional iron supplements complied less than those who were on a gastric delivery system, indicating that side effects actually did affect compliance [23].

We were surprised to find that formal employment was associated with non adherence, considering the fact that these are usually women of higher education status and better socio-economic status. We would ideally expect these mothers to be better informed about the supplements and be in a better position to buy more drugs in case stocks run out. While our findings are in agreement with those of Nordeng [8], they are contradictory to those of Muture in his study of adherence to anti- TB drugs [11, 24]. It is possible that mothers in formal employment are very busy and would frequently forget to take the iron supplements. We found no association between age, parity, marital status and adherence.

### Study limitations

This study used the visual analogue scale which allows patients to score their adherence in a range of 0–100 without being observed. This may have led to over estimation of adherence to iron supplements. This study assessed adherence for over 30 days yet in some cases the participants were received a supply of less than that in the hospital pharmacy. The patients are usually given a prescription by the attending clinician and asked to buy from private pharmacy when supply is inadequate. We feel this may not have had a very big impact since a month’s supply of antenatal iron supplements cost less than a dollar which is usually affordable by most mothers.

Assessing adherence by measuring serum concentrations of radiologically labeled iron supplements would have been a more objective way of measuring adherence. However this is very costly and may not feasible in studies with large number of participants.

## Conclusion

The level of adherence to iron supplements among antenatal mothers in Mulago National Referral Hospital was low. The factors that were independently associated with adherence were: attendance of ANC four times or more, having more than 2 weeks supply of iron supplements, prior health education and informal education of the women. There’s a need to ensure adequate drug supplies in health facilities and provision of adequate information about the supplements especially the benefits, side effects and how mothers can cope with these.

## Abbreviations

ANC: antenatal clinic
Anti TB drugs: antituberculosis drugs
ARVs: antiretroviral drugs
VAS: visual analogue scale
WHO: World Health Organisation
